# Impact of strategic use of antiretroviral therapy intervention to the HIV continuum of care in 13 cities in Indonesia: an interrupted time series analysis

**DOI:** 10.1186/s12981-021-00340-4

**Published:** 2021-04-26

**Authors:** Yane N. Tarigan, Richard J. Woodman, Emma R. Miller, Rudi Wisaksana, Paul R. Ward

**Affiliations:** 1 Health Sciences Building, College of Medicine and Public Health, Flinders University, Sturt Rd Bedford Park, Adelaide, SA 5042 Australia; 2 Department of Internal Medicine, Hasan Sadikin Hospital, University of Padjajaran, Bandung, Indonesia

**Keywords:** HIV, Continuum of care, Interrupted time series, Indonesia, TasP

## Abstract

**Background:**

In 2013 the Indonesian government introduced the strategic use of antiretroviral therapy (SUFA) initiative of expanding access to HIV test and treatment, to help achieve the UNAIDS 90–90–90 targets. However, there has been no comprehensive evaluation of the impact of this intervention in Indonesia. We conducted an interrupted time series (ITS) analysis across 6-years to assess its immediate and medium-term impact.

**Methods:**

Monthly aggregated HIV data from all HIV care clinics for persons aged ≥ 15 years were collected from 13 pilot cities. The data period encompassed 3-years prior to SUFA (26 Dec 2010–25 Dec 2013) and 3-years post-SUFA (26 Dec 2013–25 Dec 2016). The ITS was performed using a multilevel negative binomial regression model to assess the immediate and trend changes in each stage of the HIV continuum of care.

**Results:**

In the pre-SUFA period, the overall coverage in the respective risk populations for HIV tests, cases, enrolments, eligible cases and ARV initiation were 1.0%, 8.6%, 98.9%, 76.9% and 75.8% respectively. In the post-SUFA period coverage was 3%, 3.8%, 98.6%, 90.3% and 81.2% respectively—with a significant increase in the median number of HIV tests, HIV cases, those eligible for ARV treatment and treatment initiation (p < 0.05 for each). The ITS analysis demonstrated immediate increases in HIV tests (IRR = 1.41, 95% CI 1.25, 1.59; p < 0.001) and an immediate decrease in detected HIV cases per person tested (IRR = 0.77, 95% CI 0.69–0.86; p < 0.001) in the month following commencement of SUFA. There was also a 3% decline in the monthly trend for HIV tests performed (IRR = 0.97; 95% CI 0.97–0.98, p < 0.001), a 1% increase for detected cases (IRR = 1.01, 95% CI 1.0–1.02, p < 0.001), and a 1% decline for treatment initiation (IRR = 0.99,95% CI 0.99–1.0 p < 0.05).

**Conclusions:**

SUFA was associated with an immediate and sustained increase in the absolute number of HIV tests performed, detected HIV cases, and close to complete coverage of detected cases that were enrolled to care and defined as eligible for treatment. However, treatment initiation remained sub-optimal. The findings of this study provide valuable information on the real-world effect of accelerating ARV utilizing Treatment as Prevention for the full HIV continuum of care in limited resource countries.

**Supplementary Information:**

The online version contains supplementary material available at 10.1186/s12981-021-00340-4.

## Background

There is compelling evidence that initiating early treatment to people living with HIV (PLHIV) can benefit individual health and reduce HIV Transmission [1–3]. The World Health Organization (WHO) therefore recommends treating all PLHIV, irrespective of their clinical or immunological condition, as an anchor for HIV prevention [4]. A key strategy in achieving this goal is to improve the HIV continuum of care that ensures PLHIV are aware of their status, linked to care, treated as soon as possible, retained in ARV treatment and virally suppressed. Optimizing antiretroviral therapy (ART) for treatment and care has reportedly substantially reduced the morbidity and mortality of HIV [5]. This has reduced AIDS-related deaths by 33% globally and the annual incidence of HIV infections by 16% in the last decade [6]. Within Indonesia, there have been similarly large expansions in access to HIV testing and treatment over the last three decades, including a six-fold increase in the number of HIV counselling and testing clinics, a three-fold increase in the number of individuals screened for HIV, and an almost three-fold increase in the number of clinics providing ART services [7].

However, progress in HIV-related outcomes as a result of more widespread treatment has only been partly mirrored in Indonesia where, despite a 27% reduction in the annual incidence of HIV infections, AIDS related deaths has increased by 60% in the last decade [6]. In addition, in 2018 the country was well below the outlined UNAIDS 90–90–90” target [8], which states that 90% of all PLHIV should be aware of their HIV status, 90% of those diagnosed with HIV should be receiving treatment, and 90% of all people receiving ARV should be virally suppressed [9]. Prior to 2013, Indonesian figures for these two first outcomes were 24%, 13% respectively and 54% of those initiated remaining in care [7,10,11].

In response to these figures, the Indonesian government introduced a new expanded HIV testing and treatment initiative. The strategic use of antiretroviral therapy (SUFA) intervention was launched by the Indonesian Ministry of Health (MOH) in 2013 with 13 pilot sites by the Indonesian Ministry of Health (2015, ‘Final report of SUFA’s consultant’) (unpublished report). The initiative consists of finding high risk people, treating eligible PLHIV using tenofovir disoproxil fumarate (TDF) based fixed-dose combination (FDC) with lamivudine and efavirenz as first line treatment and retaining them in care. SUFA was designed to improve engagement of PLHIV within the whole HIV continuum of care and in the long term is expected to contribute to a reduction of HIV transmission. The basic framework of the overall SUFA strategy was the integrated service delivery model (LKB or *Layanan Komprehensif Berkesinambungan* in Bahasa) which builds and expands upon the existing involvement of community support organizations in improving pre- and post-ART linkages [12]. LKB refers to HIV and STI management and service delivery covering a continuum of promotive, preventive, curative and rehabilitative steps, which is provided for clients at home, in communities or in health facilities from pre-infection to the terminal stage [13]. SUFA includes partnership with HIV stakeholders, training for providers, an improved HIV testing strategy, treatment counselling, Treatment as Prevention (TasP) for specific populations, and a simplified ARV drug regimen [10].

Whilst the effectiveness of multiple interventions in individual aspects of the HIV continuum of care has been assessed in Sub-Saharan Africa and China [14–20], no studies have assessed the effect of TasP interventions on the HIV continuum of care from population testing to the clinical treatment stage within the Southeast Asia region. Cross-sectional evaluations of separate components of SUFA have been undertaken by—the Indonesian Ministry of Health (2015,’Final report of SUFA’s consultant’, (unpublished report), and 2014, ‘Mission report on monitoring and evaluation of strategic use of ART (SUFA) programme in Indonesia’ (unpublished report)), however no studies to date have provided a comprehensive long term follow-up evaluation of SUFA.

We undertook an interrupted time series (ITS) study to evaluate the effectiveness of SUFA in changing the five components in the HIV continuum of care, including HIV testing, case detection, enrolment to care, eligibility for ARV and treatment initiation.

## Methods

### Study design and setting

We used aggregated routinely collected administrative count data from the 13 pilot sites for the 3 years immediately before and after SUFA and implemented an ITS analysis, a recognized study design for assessing the effectiveness of population level public health interventions [21, 22].

The ITS design is suitable for the assessment of an intervention’s impact when using aggregated observational data collected over time. It is designed to determine the extent to which there is any “interruption” to a series of data collected over time in terms of either immediate shifts or gradual changes in the outcome, from the date at which the intervention is delivered [23–25].

The 13 pilot demonstration sites were purposely selected as being representative of the 141 HIV testing/treatment facilities across Indonesia [26]. The implementation window was from mid-December 2013 to mid-January 2014. In regards to the analysis, persons that were tested, detected for HIV, enrolled, considered eligible and initiated at any time from 26 December 2010 to 25 December 2013 were considered to be unexposed and those tested from 26 December 2013 to 25 December 2016 were considered to be exposed to the intervention. For the purposes of analysis which was performed using aggregated monthly data, we chose the last month of data collection for “non-exposure” as December 2013 and the first month that was considered as “exposed” was January 2014, since it was possible that all sites may have implemented the intervention by the end of December 2013”.

### The SUFA intervention

The details of SUFA intervention and the difference strategy between pre and post SUFA period have been fully described previously [27]. To our knowledge, there were no changes in diagnostic testing procedures or any other HIV policy programs introduced at the same time as SUFA in Indonesia, particularly in the 13 locations. The detailed strategy relevant to this study are summarized below.

The introduction and implementation of the SUFA in the sites were similar across all sites, occurring between mid-December 2013 and mid-January 2014. It involved a series of key activities and strategies (the Indonesian Ministry of Health, 2015,’Final report of SUFA’s consultant’, (unpublished report)) beginning with training in the standardized integrated service delivery model for key HIV stakeholders (local governments bodies, health providers and HIV community representatives in the regencies/cities). Following this, two days of a standardized SUFA workshop was held with attendance of key HIV stakeholders in order to build HIV networking groups, develop regency/city plans to accelerate the SUFA intervention strategy and to train regency/city facilitators [12].

### Outcomes

We assessed five HIV continuum of care outcomes as the count and rate per month, and as the rate per month per 100 persons of the population at risk for: (1) HIV tests performed, (2) newly detected HIV cases, (3) enrolment in care, (4) eligibility for ARV and (5) treatment initiation.

### Study data

The national office in the MOH Sub Directorate HIV AIDS and STI (sexually transmitted infection) in Jakarta receives monthly reports of HIV tests and treatment care report programs from the main health care facilities (ART sites) across Indonesia. Health care facility staff transfer these to the ‘reporting recording’ (RR) online database system. These electronic data were obtained for the 13 demonstration sites and included aggregated monthly population-level data for individuals aged 15 years and above. Monthly data were obtained from each site for the number of persons undergoing HIV testing, HIV cases detected, enrollments in care, cases eligible for ARV, and those initiated on treatment. Data from 26 December 2010 to 25 December 2013 were represented the pre-SUFA period and data collected from 26 December 2013 to 25 December 2016 the post-SUFA period.

### Statistical analysis

Data for the pre- and post-SUFA periods were described using the median and inter-quartile range (IQR) of the monthly counts and the two periods were compared using a Mann–Whitney test.

The intervention impacts were measured through changes in level and slope of the regression lines following the intervention. For each outcome, the ITS approach was used to assess both the ‘level change’ (the immediate impact of SUFA) and the ‘slope change’ (gradual changes of the gradient of the trend of outcomes between pre and post-SUFA until 3 years implementation). Multilevel negative binomial regression models were used to model the monthly data which accounted for the clustering of data within sites and the over-dispersion of the data. Using this model, the intercept (baseline count) is able to vary between the different sites [28], providing a specific site-level estimate of the intervention for each site and also enabled visualisation of the comparison of SUFA effects across the 13 sites.

Each model included the SUFA intervention, Time, and a Time X SUFA interaction as fixed effects, and site as a random intercept. The models provided four fixed effect parameters: 1) β_0_, the regression model constant (the mean count pre-SUFA), 2) β_1_, the pre-SUFA intervention slope across time, 3) β_2_, the change in level at the start of the implementation intervention (SUFA) and 4) β_3_, the change in slope between pre- and post-intervention (Time x SUFA intervention interaction). The post-SUFA slope was determined by adding β_1_ and β_3_. Effect estimates reported included incidence rate ratios (IRR) with 95% confidence intervals (95% CIs) and p-value based on a Wald test. As a sensitivity analysis we also included SUFA as a random slope across sites in order to test whether this changed the overall effects for the level and slope change.

We considered the effect of SUFA using counts per month (rate) (Model 1), and the rate per 100 persons at risk (Model 2). The number at risk was usually the monthly count from the preceding stage, except for HIV tests, for which the population at risk was an estimate of the key affected population (KAP) of each site. Line plots were produced to visualize the predicted level change and monthly trends for each of the 13 sites as well as the overall mean trend. Any missing data were likely to have occurred randomly due to reporting or recording issues, such as delays or failures in sending monthly reports, high staff turnover, postponement of entering data, or report completion [the Indonesian Ministry of Health, 2015, ‘Final report of SUFA’s consultant’, (unpublished report)]. Data were therefore considered to be missing at random (MAR) and unlikely to produce biased effects in the multilevel models. Bar graphs of the yearly counts for each stage provided a visual display of the overall completeness of care provided between stages. A 2-sided p-value < 0.05 was considered as statistically significant and all analyses was conducted using Stata (StataCorp, 2017. *Stata Statistical Software: Release 15*. College Station, TX: StataCorp LLC.).

## Results

During the 6-year study period, a total of 1,050,621 HIV tests were performed with 48,213 newly detected HIV cases across the 13 sites (Table 1). Amongst these cases, 40,238 were linked to HIV care, 33,654 were eligible for ARV and 24,530 were initiated for ARV. There was a significant difference in the median monthly counts for each of the five continuum care steps between pre and post SUFA.

**Table 1:** Total persons and median (IQR) monthly counts and rates for the 5 stages of HIV care across the 13 sites and 72 months of data collection (N = 936 data points)

	Total persons (N)	Monthly counts^a^		Monthly rates^b^		p-value*	
		Pre-SUFA (N = 468)	Post-SUFA (N = 468)	Pre-SUFA	Post-SUFA	^1^	^2^
HIV tests, median (IQR)	1,050,621	448 (200–910)	1546 (677–2395)	1 (.04–2)	3(1.6–8.3)	< 0.001	< 0.001
HIV cases, median (IQR)	48,213	44 (22–70)	50 (27–80)	8.6 (6.25–13.7)	3.8 (2.6–5.2)	< 0.05	< 0.001
Enrolled for care, median (IQR)	40,238	47 (24–72)	57 (27–86)	98.9 (75–116)	98.6 (78.3–121)	< 0.05	0.238
Eligible for care, median (IQR)	33,654	37 (19–53)	52 (24–74)	76.9 (66.1–89.8)	90.3 (77–100)	< 0.001	< 0.001
ARV treatment initiated, median (IQR)	24,530	23 (14–39)	39 ( 20–57)	75.8 (54.7–92)	81.2 (64.2–93.4)	< 0.001	< 0.05

^*^For pre versus post-SUFA using Mann–Whitney U test

**Fig. 1:**
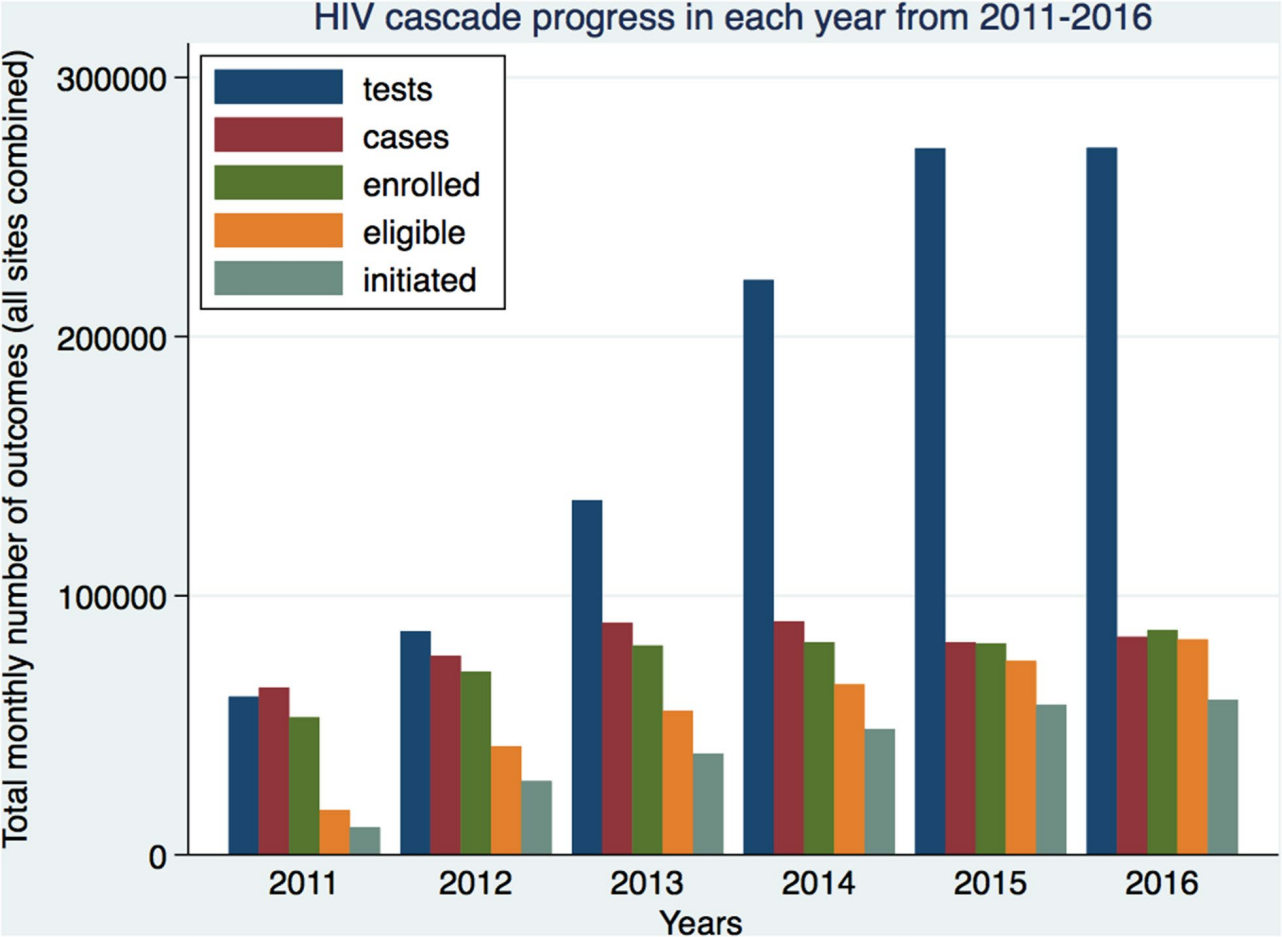
Yearly counts of each cascade of care showing the cascade progress between 2011 and 2016 (data in cases, enrolled, eligible and initiated were multiplied by 10)

The overall yearly progress of HIV continuum of care can be seen in the narrowing of the difference in bar heights between each care stage following the implementation of SUFA (see Fig. 1). However, there remained a large gap between eligibility for ART and ART initiation, highlighting a remaining challenge in achieving the 90–90–90 target.

### HIV tests

**Fig. 2:**
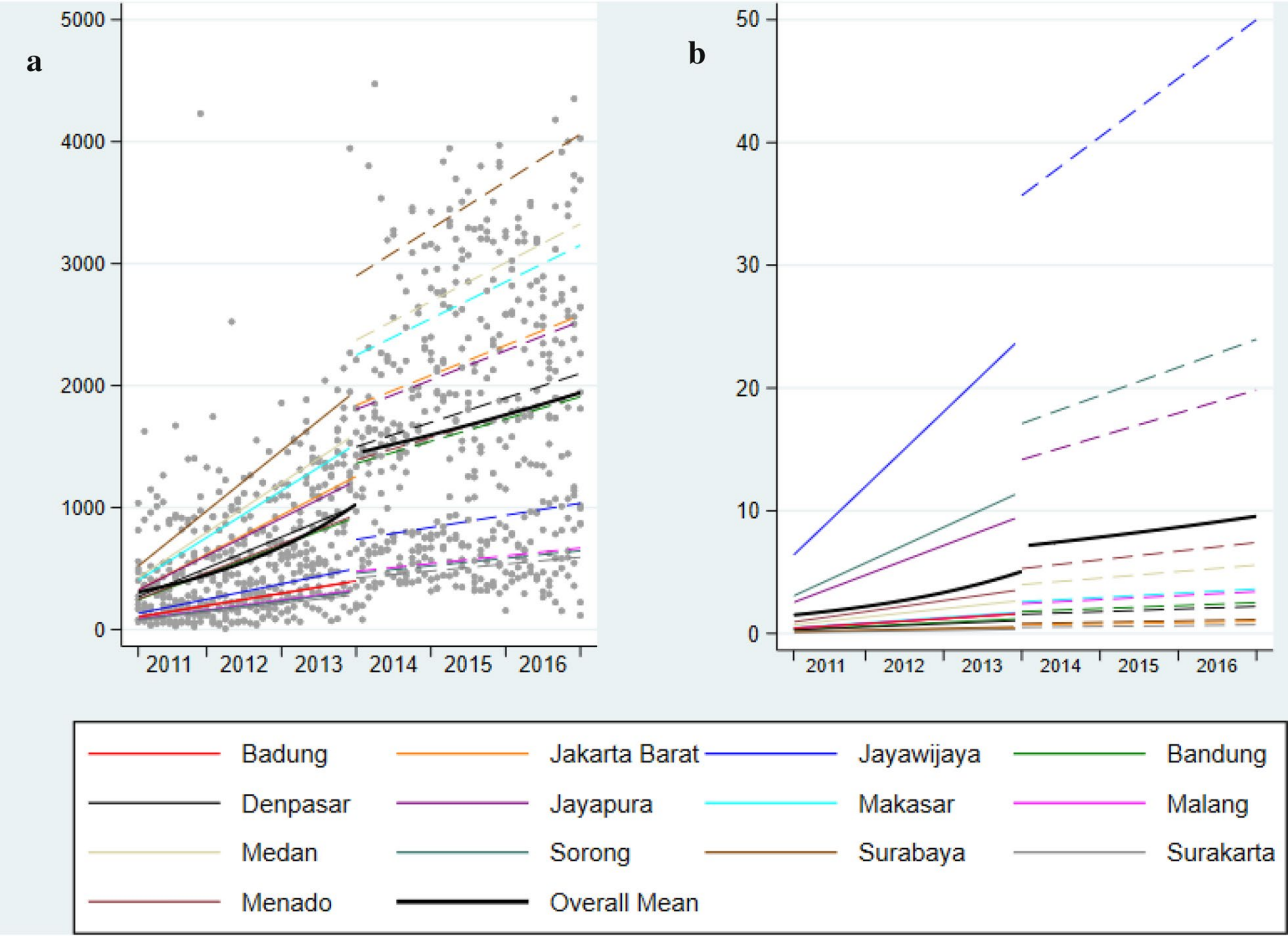
**a** Observed and predicted number of HIV tests performed each month. **b** Rate of HIV tests performed per month (persons per 100 of the estimated key affected population for each district in the same month)

Table 2 and Fig. 2 describe the rate of HIV testing pre- and post-SUFA, and the level change and slope change following the implementation of SUFA. There was a significant 41% level increase following the introduction of the SUFA and the monthly rates of HIV testing changed from a 4% increase pre-SUFA to a-less-than 1% increase post-SUFA (p < 0.001 for time x SUFA interaction).

**Table 2:** Level change^a^, pre and post SUFA rates, and the slope change^b^ for HIV tests at the point of SUFA implementation

		Model 1		Time interaction^d^	Model 2		Time interaction^d^
		IRR (95% CI)	p-value^c^		IRR (95% CI)	p-value^c^	
Level change	Predicted tests (n)						
Pre-SUFA	1024 (Dec 2013)	1.00			1.00		
Post-SUFA	1454 (Jan 2014)	1.41 (1.25, 1.59)	< 0.001		1.41 (1.25, 1.59)	< 0.001	
Slope	Predicted tests/month (n)						
Pre-SUFA (per month)	596 (Jan 2011–Dec 2013)	1.04 (1.03, 1.04)	< 0.001		1.04 (1.03, 1.04)	< 0.001	
Post-SUFA (per month)	1685 (Jan 2014–Dec 2016)	1.01 (1.00, 1.01)	< 0.001		1.01 (1.00, 1.01)	< 0.001	
Slope change							
Time × SUFA		0.97 (0.97–0.98)		< 0.001	0.97 (0.97–0.98)		< 0.001

*IRR* Incidence rate ratio

^a^Level change assesses the relative change in tests per month immediately post SUFA intervention

^b^Slope change tests the relative change in the monthly trend between pre and post SUFA. Model 1 includes fixed effects for SUFA, time and SUFA x time interaction and a random effect for site. Model 2 = Model 1 + additional adjustment for population at risk (the estimated KAP for each district site).

^c^ Estimated using a mixed effects negative binomial regression model

^d^P-value for the SUFA x Time interaction

### HIV cases

**Fig. 3:**
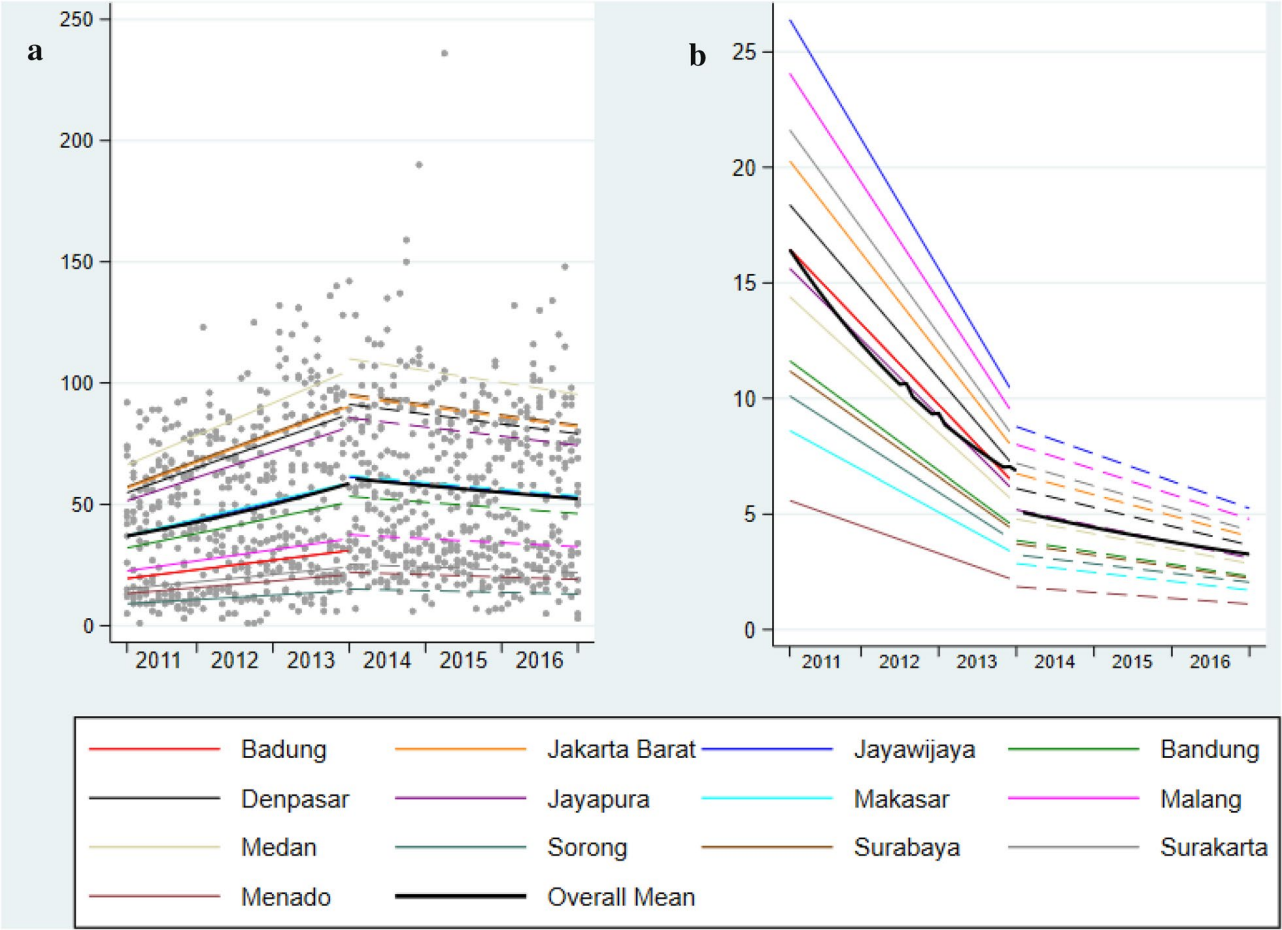
**a** Observed and predicted number of HIV cases detected each month. **b** Rate of HIV cases detected per month (persons per 100 of the district HIV tests performed in the same month)

Table 3 and Fig. 3 present the level and slope change for the rate of HIV case detection pre- and post-SUFA. The absolute number of cases detected (model 1) did not change post-SUFA. However, after adjusting for the number of HIV tests performed per month (model 2), there was a 23% level decrease in detected HIV cases per HIV test performed. In addition, there was a significant slope decrease between the two SUFA periods (p < 0.001 for time X SUFA interaction) (from approximately 1% pre SUFA to less than 1% post-SUFA). The declining rate in HIV cases detected per HIV test of ~ 3% pre-SUFA changed to a declining rate of < 1% post-SUFA (p < 0.001).

**Table 3:** Level change^a^, pre and post SUFA rates, and the slope change^b^ for HIV cases at the point of SUFA implementation

		Model 1		Time interaction^d^	Model 2		Time interaction^d^
		IRR (95% CI)	p-value^c^		IRR (95% CI)	p-value^c^	
Level change	Predicted cases (n)						
Pre-SUFA	58.7 (Dec 2013)	1.00			1.00		
Post-SUFA	60.5 (Jan 2014)	1.04 (0.95, 1.13)	0.445		0.77 (0.69, 0.86)	< 0.001	
Slope	Predicted cases/month (n)						
Pre-SUFA (per month)	47.1 (Jan 2011-Dec 2013)	1.01 (1.01, 1.02)	< 0.001		0.97 (0.97, 0.98)	< 0.001	
Post-SUFA (per month)	56.3 (Jan 2014-Dec 2016)	1.00 (1.00, 1.00)	< 0.001		0.99 (0.98, 0.99)	< 0.001	
Slope change							
Time × SUFA		0.98 (0.98–0.99)		< 0.001	1.01 (1.01–1.02)		< 0.001

* IRR* Incidence rate ratio

^a^Level change assesses the relative change in tests per month immediately post SUFA intervention

. ^b^Slope change tests the relative change in the monthly trend between pre and post SUFA. Model 1 includes fixed effects for SUFA, time and SUFA x time interaction and a random effect for site. Model 2 = Model 1 + additional adjustment for population at risk (the HIV tests performed for each district site)

^c^Estimated using a mixed effects negative binomial regression model

. ^d^P-value for the SUFA x Time interaction

### Enrolment to care

Additional file 1: Table S1 and Figure S1 show level and slope changes in the rates of enrolment to care pre- and post-SUFA. There were no level changes following the introduction of SUFA. However, there was a slope decline from ~ 2% pre-SUFA period to an approximately flat trend post-SUFA period. In addition, the slope for the rate of enrolments to care per month per detected HIV case changed from a + 1% per month increase pre-SUFA to a -1% trend post SUFA (p < 0.001).

Additional file 1: Table S2 and Figure S2 present the level and slope change for the rate of eligibility for ARV pre- and post-SUFA. There were no level changes following the introduction of SUFA. The trend in the absolute number eligible for ARV per month decreased slightly from a 2% per month increase to a 1% per month increase. However, there was no change in slopes for the monthly rate per HIV persons enrolled (p = 0.499).

### Treatment initiation

Additional file 1: Table S3 and Figure S3 describe level and slope change for the rate of treatment initiation pre- and post-SUFA. There was no level change in either the absolute rate or the rate per eligible HIV person following the SUFA introduction. The trend in the absolute rate of treatments initiated changed from an increase of 2% pre SUFA to approximately 1% post SUFA (p < 0.001). There was also a significant change in the rate of treatment initiations per eligible case which changed from < 1% increase pre SUFA to a slow decline of 1% (p < 0.05).

### Sensitivity analysis

When SUFA was included in the models as a random slope across the 13 sites, the results for all outcomes were similar and none of the findings changed substantively (data not shown).

## Discussion

To our knowledge, this study is the first to assess the impact of expanding access to HIV tests and ARV treatment utilising criteria of irrespective CD4 count for a specific population on the HIV continuum of care in a Southeast Asian country using an ITS approach. Our results indicate an overall increase in the median number of HIV tests, HIV cases, eligibility for ARV and treatment initiation. In addition, the ITS analysis demonstrated immediate increases in HIV testing and decreases in detected HIV cases as well as significant trend changes for tests, cases, enrolment to care, and eligibility for ARV treatment. A previous MOH monitoring and evaluation report conducted in the same areas also found an overall increase in the number of people engaged in HIV testing, the HIV detection rate, enrolment to HIV care, and treatment initiation [Indonesian Ministry of Health, 2014, ‘Mission report on monitoring and evaluation of strategic use of ART (SUFA) programme in Indonesia’ (unpublished report)] from 3-years prior to 1-year post SUFA but did not assess the immediate versus longer term impacts.

Studies in lower-to-middle income countries investigating the effectiveness of structural interventions in expanding access to HIV tests and ART and strengthening the HIV cascade pathway have been in mostly hyper-endemic countries in the Sub-Saharan Africa region [15, 16, 18, 29–38]. In addition, studies have generally evaluated effectiveness for either a single or limited number of the specified stages rather than multiple care stages simultaneously [29, 32, 33]. Although two studies have reported on epidemic settings in Asia, none have assessed Southeast Asia [19, 20] particularly utilising TasP intervention. Whilst the aggregate effect of interventions has been assessed using repeated cross-sectional data, the temporal (i.e. level and slope) changes have not previously been examined [37].

Our study identified an immediate increased level but decreased trend in HIV tests performed each month following the introduction of SUFA, indicating an immediate impact and a continuation in the improvements in testing rates, albeit at a slightly reduced rate. This reduction in the trend may be related to overall better adoption of the initiatives over time [39] for the high-risk community. In North Carolina, there was a similar change in the monthly trend of HIV tests following the adoption of opt-out for routine HIV testing (PICT) and case detection. Whilst HIV tests continued to increase (34 tests per month and 0.46 tests per 100,000 persons per month), the rate of increase was slightly flatter than prior to the intervention (55 tests per month and 0.81 tests per 100 000 persons per month) [40]. Our findings support the MOH evaluation, which reported upward trends in HIV testing and counselling in the thirteen districts [the Indonesian Ministry of Health, 2015,’Final report of SUFA’s consultant’, (unpublished report)].

We observed a significant increase in the rate of eligibility for ARV treatment. Contributing factors include an increase in the absolute number of enrolments to care each month as well as the absolute number of people assessed as being eligible due to the expanded eligibility criteria, as shown by the data for median enrolment and eligibility for ARV. There was also a slower increase in the rate of eligibility for ARV which might be explained by the proportion eligible at the end of pre-SUFA having already reached a relatively high level, with a limit to further possible increases. However, greater increases with the expanded SUFA eligibility criteria could still have occurred if infected persons were identified earlier, allowing for earlier determination of eligibility for ARV and earlier initiation of treatment. In a SUFA consultative document, it was proposed that expansion of the treatment criteria could double the number of people eligible for ARV, which could also increase those receiving treatment [10]. We considered increased infrastructure and human resources associated with the SUFA intervention to be the other main contributors to the rapid expansion of access to counselling and testing. These include campaigns promoting HIV tests, outreach programs, establishment of new HIV testing service clinics, increases in staff (within and outside hospitals and clinics) with capacity to provide PICT and VCT, promotion of SUFA to professional organisations including the Indonesian Midwives Association, and expanded networking among clinics, hospitals, lay workers, community based organisations, key affected populations and subdistrict task forces [the Indonesian Ministry of Health, 2015, ‘Final report of SUFA’s consultant’, (unpublished report)), and Ministry of Health, 2014 ‘Monthly report from Medan City ‘ (Unpublished report)].

We observed a decline in both the level and slope for the rate of HIV cases detected per HIV test. This might be explained by either differential regional HIV prevalence, performance of the HIV testing programs, or individual readiness to be diagnosed [19]. More likely is the disproportionate targeting in the SUFA areas from higher to lower risk groups, resulting in fewer cases per test. A specific policy to expand HIV tests to pregnant women was suggested in 2013 [41] and was subsequently incorporated into the SUFA intervention. Pregnant women are easier to reach relative to key affected population (KAP) as they already access clinic care. KAPs refers to groups who have specific higher risk behaviour that makes them vulnerable to acquiring HIV-AIDS regardless of epidemic level and local context [42].

In contrast, the KAP involves sizeable human effort, time, logistics and money. Thus, whilst pregnant mothers might be considered a lower risk group, it may have impacted on the rate of HIV case detection [41], whereas expansion of testing in KAPs has a less substantial impact [Indonesian Ministry of Health, 2014, ‘Mission report on monitoring and evaluation of strategic use of ART (SUFA) programme in Indonesia’ (unpublished report)].

The trend for enrolments to care declined slightly following SUFA, which may partly reflect the reducing trend in the absolute number of newly diagnosed HIV cases. However, a small proportion of newly diagnosed HIV cases may not have been linked to care post-SUFA since expansion of testing centres to within and outside hospitals/clinics might have created somewhat immature referral systems and tracking mechanisms. Clinics that only have HIV testing services without offering HIV care and treatment are permitted by Indonesian regulation as long as they refer the patients to centres with more comprehensive care and treatment capabilities [43]. Since the median proportion of enrolments in care in the pre-SUFA (98.9%) were very high and similar to post-SUFA (98.6%), almost all newly HIV detected cases were already being and continued to be successfully linked to care. The availability of ‘facility based HIV Testing and Counselling (VCT or PICT)’ in both periods might explain this, especially given HIV testing is predominantly conducted in facilities with comprehensive HIV care and treatment [44]. A HIV continuum of care cohort study conducted in four locations in Indonesia supports the theory that being tested and treated in the same health care facility is an important predictor for enrolling into HIV care [45].

The trend in the treatment initiations and the rate of treatment initiations per person eligible for ARV per month was flatter post-SUFA but continued to increase, reflecting both the increased number of people eligible for ARV as well as the increase in the number of persons initiated for ARV, where the median increased substantially from 23 to 38.5. The expansion of Highly Active ART in South Africa including the decentralization of care to primary health centers, increased staff involvement and improved teamwork also improved treatment initiation [46]. Additionally, TasP interventions increased treatment initiation in Sub-Saharan Africa and China [16, 19, 30, 36, 38]. Despite this, the median proportion of treatment initiations increased only marginally from 77.8 to 81.2% suggesting that about 20% of people eligible did not obtain treatment in either period. This was despite simplification of the treatment procedure for compulsory CD4 counts and, if indicated and available, the frequency of treatment adherence counselling from being a subjective number to four times/year [43]. A recent scoping review conducted in Indonesia found that economic problems related to CD4 testing and blood examination, administration and transportation costs, and fear of treatment side effects [47] were some of the reasons for not receiving treatment.

SUFA was designed as a combination of interventions to tackle multiple levels of drivers of HIV control and management in Indonesia [48]. There was broad similarity of the 13 sites based on the entry criteria i.e. having already employed the LKB model, a high HIV program burden (HIV prevalence, as shown by the number of key affected population reaching at least 200), good support from their respective internal health service system infrastructures, and the availability of non-government organisations. Further, the selected regencies/cities had support from the HIV community and had demonstrated a commitment to contribute to the program expenditure [12]. However, variation in the contextual factors such as human and financial resources, geographical, the urban versus rural divide, socio demographic, economic, cultural and religious factors which also contributed to HIV transmission level, program performance and size of the site, might be important in determining the sometimes large variation in effects across the 13 sites.

Our study had several strengths including the use of ITS multilevel models, which allowed assessment of both level and slope changes. In addition, we performed a comprehensive assessment of the intervention, assessing all stages in the HIV continuum of care, from rates of HIV testing to ARV treatment. The use of a large dataset with 13 sites and 6 years of data for each care stage allowed accurate assessment of the likely impact across Indonesia.

There were also some limitations. Given the aggregated nature of the data, we were unable to adjust for age and gender or other demographic factors. However, this is unlikely given that population level changes, even within the high-risk HIV population, are unlikely to have changed significantly over time. We were also unable to completely capture all data across the 13 sites and 72 months. However, we are confident that the missing data was of a random nature, and is therefore unlikely to cause significant bias even if there was varying degrees of missingness across sites.

Information on the availability of ART in the two era is important for helping determine the reasons for success of SUFA, we unfortunately do not have this information. An assumption therefore is that there was no shortages of ART supply in the two periods. This is a reasonable assumption since there was no funding interruption reported from 2007 onwards [49]. In addition, individual level data including that on the risk group would have reduced the possibility of unmeasured confounding and allowed a valuable understanding of possible heterogeneity of the SUFA effect in different risk groups. However, our previous publication in a smaller number of sites did not suggest any major variation in the treatment effect of SUFA across the different risk groups [27].

However the MOH report on the monitoring and evaluation of SUFA, provides some information that imbalanced population targeting of high risk populations might have occurred. The report indicated that, although HIV testing and counselling increased significantly from 2010–2014, this was likely due to an increase of testing of pregnant mothers rather than of key affected populations, particularly between 2013–2014 in the thirteen sites as we have previously explained.

## Conclusion

The impetus for the SUFA project was the postulation that unless substantial changes in HIV testing and treatment were made, the ultimate goals of reductions in morbidity, mortality and HIV transmission would be threatened. This study provides important information on the performance of the Indonesian health system in adopting the SUFA policy strategy that included the utilization of TasP. We have described the strengths of the policy and its effectiveness in increasing the number of people engaged in most steps of the HIV continuum of care cascade. and the challenges faced by the Indonesian health system. Likewise, although this study did not specifically assess the 90–90-90- achievement of SUFA, our findings clearly show that work remains if Indonesia is to achieve the UNAIDS 90–90–90 goal by 2020, and the UNAIDS 95–95–95 goal by 2030 [50].

## Supplementary Information

**Additional file 1. Table S1.** Level change^1^, pre and post SUFA rates, and the slope change^2^ for enrolment to care at the point of SUFA implementation**. Figure S1.** A) Observed and predicted number of persons enrolled to care each month. B) Rate of enrolments per month (persons per 100 of the district HIV cases in the same month). **Table S2.** Level change^1^, pre and post SUFA rates, and the slope change^2^ for eligibility for ARV at the point of SUFA implementation. **Figure S2.** A) Observed and predicted number of persons eligible for ARV each month. B) Rate of ARV eligibility per month (persons per 100 of the district enrolments in the same month). Estimated rate sometimes exceeds 100 eligible per 100 persons enrolled due to total of eligible in one month being higher than the previous month's total for enrolments persons. **Table S3.** Level change^1^, pre and post SUFA rates, and the slope change^2^ for ARV initiated at the point of SUFA implementation. **Figure S3.** A) Observed and predicted number of persons initiated for ARV each month. B) Rate of ARV initiation per month (persons per 100 of the district ARV eligible persons in the same month).

## Data Availability

The data that supported the study are sourced from the Indonesian Ministry of Health at the national office. The data are available in the HIV AIDS information system (Sistem informasi HIV-AIDS), but there may be restrictions on access to the data since the use of these data was permitted exclusively for this study. The data, therefore, is not publicly available. However, upon a reasonable request and with the permission of the Ministry of Health, data may be shared by the authors.
