# Supplementary material for: Impact of strategic use of antiretroviral therapy intervention to the HIV continuum of care in 13 cities in Indonesia: an interrupted time series analysis
Source: AIDS Res Ther. 2021 Apr 26;18:22. doi: 10.1186/s12981-021-00340-4 (PMC8074419; doi:10.1186/s12981-021-00340-4)
Supplement: Supplementary file 1 — Additional file 1. Table S1. Level change1, pre and post SUFA rates, and the slope change2 for enrolment to care at the point of SUFA implementation. Figure S1. A) Observed and predicted number of persons enrolled to care each month. B) Rate of enrolments per month (persons per 100 of the district HIV cases in the same month). Table S2. Level change1, pre and post SUFA rates, and the slope change2 for eligibility for ARV at the point of SUFA implementation. Figure S2. A) Observed and predicted number of persons eligible for ARV each month. B) Rate of ARV eligibility per month (persons per 100 of the district enrolments in the same month). Estimated rate sometimes exceeds 100 eligible per 100 persons enrolled due to total of eligible in one month being higher than the previous month's total for enrolments persons. Table S3. Level change1, pre and post SUFA rates, and the slope change2 for ARV initiated at the point of SUFA implementation. Figure S3. A) Observed and predicted number of persons initiated for ARV each month. B) Rate of ARV initiation per month (persons per 100 of the district ARV eligible persons in the same month). [file 12981_2021_340_MOESM1_ESM.docx]

**Additional File 1**

Table S1. Level change^1^, pre and post SUFA rates, and the slope change^2^ for enrolment to care at the point of SUFA implementation.

|  |  | **Model 1** | | **Time interaction^5^** | **Model 2** | | **Time interaction^5^** |
| --- | --- | --- | --- | --- | --- | --- | --- |
|  |  | **IRR^3^ (95 % CI)** | **p-value^4^** |  | **IRR (95 % CI)** | **p-value^4^** |  |
| ***Level change*** | *Predicted enrolment (n)* |  |  |  |  |  |  |
| Pre-SUFA | 61.6 (Dec 2013) | 1.00 |  |  | 1.00 |  |  |
| Post-SUFA | 65.0 (Jan 2014) | 1.06 (0.93, 1.21) | 0.391 |  | 1.12 (0.97, 1.29) | 0.119 |  |
| ***Slope*** | Predicted enrolment/month (n) |  |  |  |  |  |  |
| Pre-SUFA (per month) | 47.3 (Jan 2011-Dec 2013) | 1.016 (1.01, 1.02) | <0.001 |  | 1.01 (1.0, 1.02) | <0.05 |  |
| Post-SUFA (per month) | 60.7 (Jan 2014-Dec 2016) | 1 (0.99, 1.00) | 0.063 |  | 0.994 (0.989, 0.998) | <0.05 |  |
| ***Slope change*** |  |  |  |  |  |  |  |
| Time x SUFA |  | 0.98 (0.97-0.99) |  | <0.001 | 0.98 (0.98-0.99) |  | <0.001 |

^1^Level change assesses the relative change in tests per month immediately post SUFA intervention. ^2^Slope change tests the relative change in the monthly trend between pre and post SUFA. Model 1 includes fixed effects for SUFA, time and SUFA x time interaction and a random effect for site. Model 2=Model 1+additional adjustment for population at risk (the HIV cases detected for each district site). ^3^IRR=Incidence rate ratio. ^4^Estimated using a mixed effects negative binomial regression model. ^5^P-value for the SUFA x Time interaction.


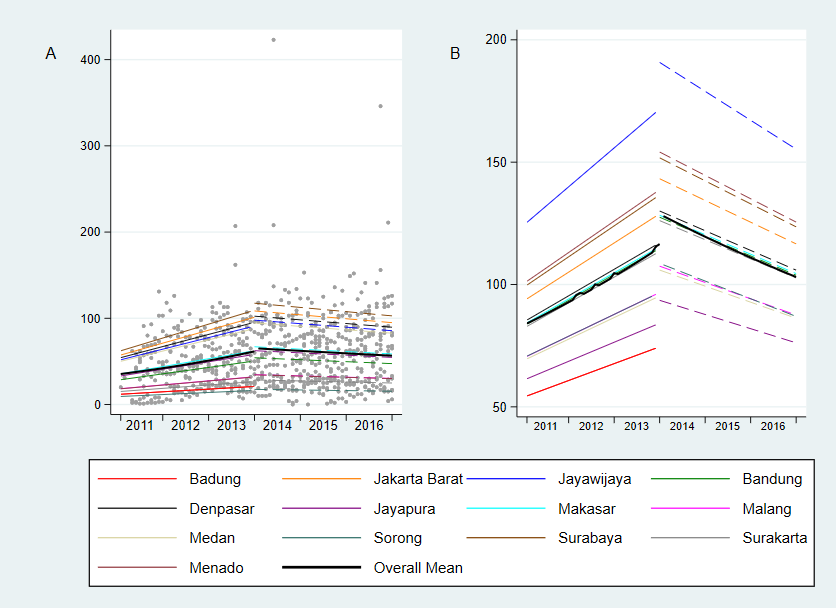


Figure S1: A) Observed and predicted number of persons enrolled to care each month. B) Rate of enrolments per month (persons per 100 of the district HIV cases in the same month).

Eligibility for ARV. Estimated rate sometimes exceeds 100 enrolled per 100 persons cases due to total of enrolments in one month being higher than the previous month's total for HIV persons.

Table S2. Level change^1^, pre and post SUFA rates, and the slope change^2^ for eligibility for ARV at the point of SUFA implementation.

|  |  | **Model 1** | | **Time interaction^5^** | **Model 2** | | **Time interaction^5^** |
| --- | --- | --- | --- | --- | --- | --- | --- |
|  |  | **IRR^3^ (95 % CI)** | **p-value^4^** |  | **IRR (95 % CI)** | **p-value^4^** |  |
| ***Level change*** | *Predicted eligibility (n)* |  |  |  |  |  |  |
| Pre-SUFA | 48.2 (Dec 2013) | 1.00 |  |  | 1.00 |  |  |
| Post-SUFA | 49.8 (Jan 2014) | 1.03 (0.91, 1.17) | 0.66 |  | 1.02 (0.93, 1.11) | 0.75 |  |
| ***Slope*** | *Predicted eligibility/month (n)* |  |  |  |  |  |  |
| Pre-SUFA (per month) | 35.7 (Jan 2011-Dec 2013) | 1.02 (1.01, 1.02) | <0.001 |  | 1.00 (1.00, 1.01) | 0.112 |  |
| Post-SUFA (per month) | 54.2 (Jan 2014-Dec 2016) | 1.01(1.001, 1.01) | <0.05 |  | 1.005(1.002, 1.008) | <0.05 |  |
| ***Slope change*** |  |  |  |  |  |  |  |
| Time x SUFA |  | 0.99 (0.98-0.99) |  | <0.001 | 1.00 (1.00-1.01) |  | 0.499 |

^1^Level change assesses the relative change in tests per month immediately post SUFA intervention. ^2^Slope change tests the relative change in the monthly trend between pre and post SUFA. Model 1 includes fixed effects for SUFA, time and SUFA x time interaction and a random effect for site. Model 2=Model 1+additional adjustment for population at risk (the enrolment to care for each district site). ^3^IRR=Incidence rate ratio. ^4^Estimated using a mixed effects negative binomial regression model. ^5^P-value for the SUFA x Time interaction.


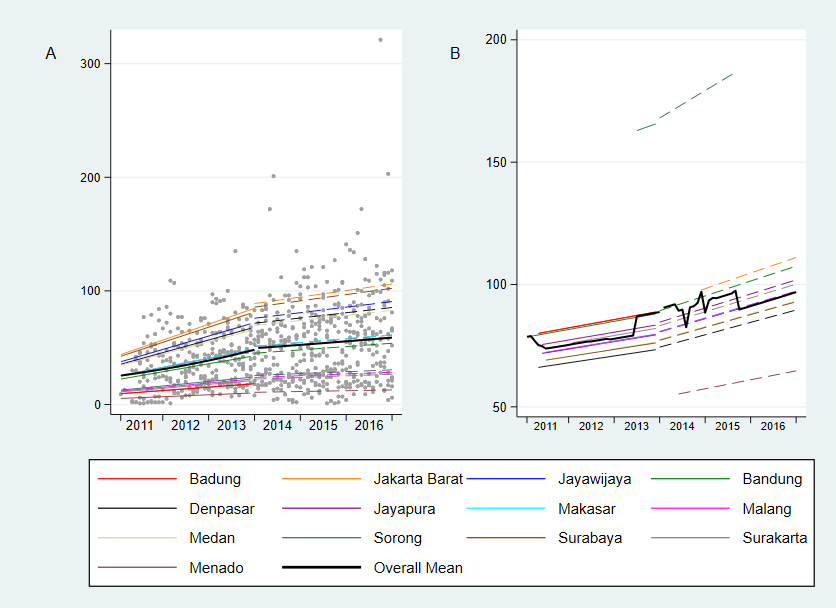


Figure S2: A) Observed and predicted number of persons eligible for ARV each month. B) Rate of ARV eligibility per month (persons per 100 of the district enrolments in the same month). Estimated rate sometimes exceeds 100 eligible per 100 persons enrolled due to total of eligible in one month being higher than the previous month's total for enrolments persons

Table S3. Level change^1^, pre and post SUFA rates, and the slope change^2^ for ARV initiated at the point of SUFA implementation.

|  |  | **Model 1** | | **Time interaction^5^** | **Model 2** | | **Time interaction^5^** |
| --- | --- | --- | --- | --- | --- | --- | --- |
|  |  | **IRR^3^ (95 % CI)** | **p-value^4^** |  | **IRR (95 % CI)** | **p-value^4^** |  |
| ***Level change*** | *Predicted treatment (n)* |  |  |  |  |  |  |
| Pre-SUFA | 34.6 (Dec 2013) | 1.00 |  |  | 1.00 |  |  |
| Post-SUFA | 37.2 (Jan 2014) | 1.07 (0.96, 1.2) | 0.241 |  | 1.09 (0.99, 1.20) | 0.089 |  |
| ***Slope*** | *Predicted treatment/month (n)* |  |  |  |  |  |  |
| Pre-SUFA (per month) | 25 (Jan 2011-Dec 2013) | 1.02 (1.02, 1.03) | <0.001 |  | 1.005 (1.00, 1.01) | <0.05 |  |
| Post-SUFA (per month) | 40 (Jan 2014-Dec 2016) | 1.01 (1.00, 1.01) | <0.05 |  | 0.998 (0.995, 1.002) | 0.323 |  |
| ***Slope change*** |  |  |  |  |  |  |  |
| Time x SUFA |  | 0.98 (0.98-0.99) |  | <0.001 | 0.99 (0.99-1.00) |  | <0.05 |

^1^Level change assesses the relative change in tests per month immediately post SUFA intervention. ^2^Slope change tests the relative change in the monthly trend between pre and post SUFA. Model 1 includes fixed effects for SUFA, time and SUFA x time interaction and a random effect for site. Model 2=Model 1+additional adjustment for population at risk (the eligibility for ARV for each district site). ^3^IRR=Incidence rate ratio. ^4^Estimated using a mixed effects negative binomial regression model. ^5^P-value for the SUFA x Time interaction.


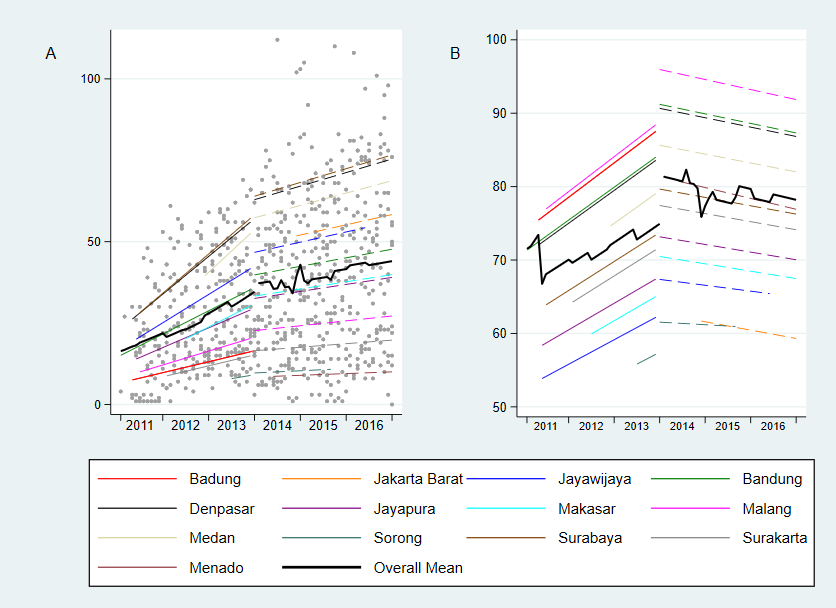


Figure S3: A) Observed and predicted number of persons initiated for ARV each month. B) Rate of ARV initiation per month (persons per 100 of the district ARV eligible persons in the same month).
